# Prostatic Artery Embolization as a Treatment Option for Symptomatic Benign Prostatic Hyperplasia: Results from the Prospective Follow-Up Study in Lithuania

**DOI:** 10.3390/medicina59101871

**Published:** 2023-10-21

**Authors:** Tautvydas Jankauskas, Edgaras Buržinskis, Rytis Stasys Kaupas, Algidas Basevičius, Mindaugas Jievaltas

**Affiliations:** 1 Radiology Clinic, Lithuanian University of Health Sciences, 44307 Kaunas, Lithuania; 2 Surgery Clinic, Lithuanian University of Health Sciences, 44307 Kaunas, Lithuania; 3 Urology Clinic, Lithuanian University of Health Sciences, 44307 Kaunas, Lithuania

**Keywords:** benign prostate hypertrophy, prostatic artery embolization, follow-up, outcomes, age

## Abstract

*Background*: The endovascular treatment of symptomatic benign prostate hypertrophy (BPH) by prostatic artery embolization (PAE) is one of the new treatments proposed. PAE is a minimally invasive alternative that has been shown to successfully treat lower urinary tract symptoms in BPH patients by causing infarction and necrosis of hyperplastic adenomatous tissue, which decompresses urethral impingement and improves obstructive symptoms. The aim of this study was to evaluate the effectiveness and efficacy of PAE in relieving symptoms in patients with symptomatic BPH. *Materials and Methods*: The material for the study was collected from 2019 to 2022. A total of 70 men with BPH and PAE were studied. Patients underwent an urological examination to measure the International Prostate Symptom Score (IPSS), Quality of Life score (QoL), International Index of Erectile Function short form (IIEF-5), uroflowmetry with Qmax, prostatic volume (PV), and post-void residual volume (PVR) measurements. Statistical analysis for dependent samples was applied. Measured parameters at 2 months and 6 months follow-up were compared to baseline. *Results:* At baseline, the age of the male (N = 70) subjects was 74 ± 9.6 years with a median of 73.8, but fluctuated from 53 to 90 years. The mean of PV was almost 111 mL and the Qmax was close to 7.7 mL/s. The average PVR was 107.6 mL. The IPSS score mean was 21.3 points and the QoL score was 4.53 points. The IIEF-5 questionnaire score was almost 1.8 points, which shows severe erectile dysfunction. The mean value of the PSA level was 5.8 ng/mL. After 2 and 6 months of PAE, all indicators and scores except erectile function significantly improved. *Conclusions:* The outcomes of our study show promising results for patients with benign prostatic hyperplasia after PAE. The main prostate-related parameters (PV, Qmax, PVR, IPSS) improved significantly 6 months after embolization.

## 1. Introduction

Benign prostatic hyperplasia (BPH) is common in men, ranging from 50% in 50-year-olds to 90% in 90-year-olds. Benign prostatic hyperplasia is frequently associated with troublesome lower urinary tract symptoms (LUTS). For decades now, transurethral resection of the prostate (TURP) has been the gold standard surgical intervention for LUTS associated with BPH [1]. 

BPH presents with lower urinary tract symptoms, mainly obstructive symptoms such as weak urine stream, incomplete bladder emptying, and frequent urinating at night [2,3]. To eliminate these symptoms, surgical interventions are usually used to improve the patient’s condition and quality of life, relieve the symptoms, and stop the progression of the disease. Most of the time, due to effective pharmacotherapy, surgical interventions are prescribed only to patients whose symptoms persist [4]. 

When surgical management is necessary, there are three general types to choose from: transurethral surgery, simple prostatectomy, and minimally invasive surgical therapies [5]. Patient evaluation must be performed to select the most optimal treatment method. Factors like prostate gland size, patient comorbidities, and medications used, especially anticoagulation therapy, should be considered [6]. For large prostate glands (>80 mL), usually prostatectomy (open, laparoscopic, or robotic) is performed, also laser enucleation of the prostate is an option. For average prostate size (30–80 mL), it is recommended to perform transurethral resection of the prostate, laser enucleation, photoselective or transurethral vaporization, prostatic urethral lift, or water vapor thermal energy. For small prostates (<30 mL), transurethral incision of the prostate and minimally invasive options from the average prostate treatment group can be used [6]. Usually, the method of choice depends on the physician itself, also on the availability of the surgical equipment in a particular hospital. New treatment options for BPH, including higher selectivity alpha-blockers, intraprostatic injections, and prostatic artery embolization are being researched and investigated [7].

Surgical treatment using TURP is the gold standard for the treatment of BPH. The patient’s age, baseline prostate volume, degree of obstruction, international prostate symptom score, peak urine flow rate, serum prostate-specific antigen level, and residual urine volume are important prognostic markers [8,9]. Despite the effectiveness and efficiency of surgical treatment, complications of the disease are quite common. These include post-operative urinary tract infection, urethral stricture, post-operative pain, urinary incontinence or retention, erectile dysfunction, and circulatory disturbance [10]. In order to avoid those complications, it was necessary to search for minimally invasive and effective treatments to improve the treatment strategy to achieve equivalent efficacy and avoid surgery-related complications [10].

One of the new treatments proposed was the endovascular treatment of symptomatic BPH by prostatic artery embolization (PAE). Currently, PAE is a minimally invasive alternative that has been shown to successfully treat LUTS in BPH patients by causing infarction and necrosis of hyperplastic adenomatous tissue, which decompresses urethral impingement and improves obstructive symptoms [3]. This treatment is similar to the treatment of uterine fibroids by embolization of uterine arteries [11]. Preliminary studies of the application of the PAE have shown promising results [12]. PAE is a complex technique with a high rate of technical failure (2–3%), and approximately 15% of patients undergo unilateral PAE due to technical difficulties [8]. In PAE, many embolic agents can be used as 300–500 μm microspheres and 150–250 μm polyvinyl alcohol particles [13].

The aim of this study was to evaluate the effectiveness and efficacy of PAE in relieving symptoms in patients with symptomatic benign prostatic hyperplasia.

## 2. Materials and Methods

### 2.1. Patients Population

The material for the study was collected from 2019 to 2022. A total of 70 men with BPH and PAE were studied. Due to the fact that not all patients with LUTS are suitable for PAE, inclusion and exclusion criteria were defined. Inclusion criteria were male patients older than 50 years with moderate to severe LUTS (IPSS > 8 points, Qmax < 12 mL/s), who were refractory to pharmacotherapy (alpha-1-adrenergic receptor antagonists or/and 5-alpha-reductase inhibitors) for at least 6 months, and whose prostatic volume was more than 60 mL. Exclusion criteria were prostatic cancer, bladder calculi, bladder diverticula, severe coagulation disorders, sensitivity to iodine, and chronic renal failure (except those who were treated with dialysis).

Patients who were suitable for PAE underwent an urological examination to measure the international prostate symptom score (IPSS) with a score range: 0–7—mildly symptomatic, 8–19—moderately symptomatic, and 20–35—severely symptomatic [14,15]; quality of life (QoL) score was measured by Likert scale, where 0 (delighted), 1 (pleased), 2 (mostly satisfied), 3 (mixed about equally satisfied and dissatisfied), 5 (mostly dissatisfied), and 6 (terrible) [16]; international index of erectile function (IIEF) was measured by a short form questionnaire (IIEF-5) and ranged from 0 to 25 (the patient would receive 0 score if he had no intercourse at all), and was classified into five categories based on the scores: severe (0–7), moderate (8–11), mild to moderate (12–16), mild (17–21), without erectile dysfunction (22–25) [17,18]; and uroflowmetry with Qmax measurement [15]. Also, transrectal ultrasound (TRUS) was performed to measure prostatic volume (PV), and pelvis ultrasound was performed to measure post-void residual volume (PVR) [15,19]. Prostatic specific-antigen (PSA) evaluation was conducted via blood investigation [20].

**Chart 1:**
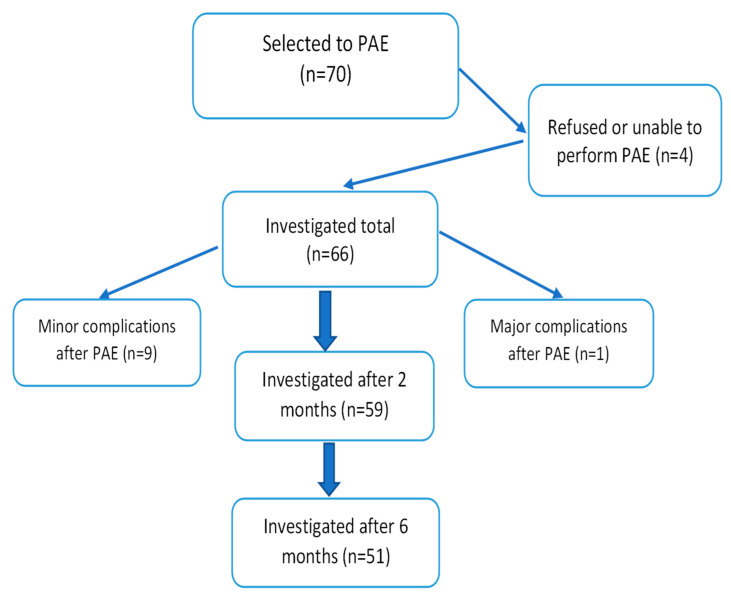
Research design.

The study design is presented in Chart 1.

The PSA density (PSAD) value was also evaluated according to the PSAD value calculation algorithm [21]. 

Some chronic disease risk factors such as body mass index (BMI), harmful lifestyle factors such as smoking and alcohol consumption (No and Yes), and sexual life as erectile function (No and Yes) were also assessed. Body mass index values are as follows: <18.5 kg/m^2^, too low; 18.5–24.9 kg/m^2^, normal; 25.0–29.9 kg/m^2^, overweight; ≥30.0 kg/m^2^, obese.

If prostatic cancer is suspected, a pelvic MRI is performed before embolization. All patients underwent the same examination routine: before the PAE procedure and then 2 months and 6 months after the procedure. Only PSA levels were measured after 6 months.

This research was approved by the Kaunas Regional Biomedical Research Ethics Committee (approval No. BE-2-68) (approved on 8 October 2018) and was performed in accordance with the Declaration of Helsinki. Informed consent was obtained from all patients for this study and all patients agreed in written form to participate in the study.

### 2.2. Embolization Technique 

**Figure 1:**
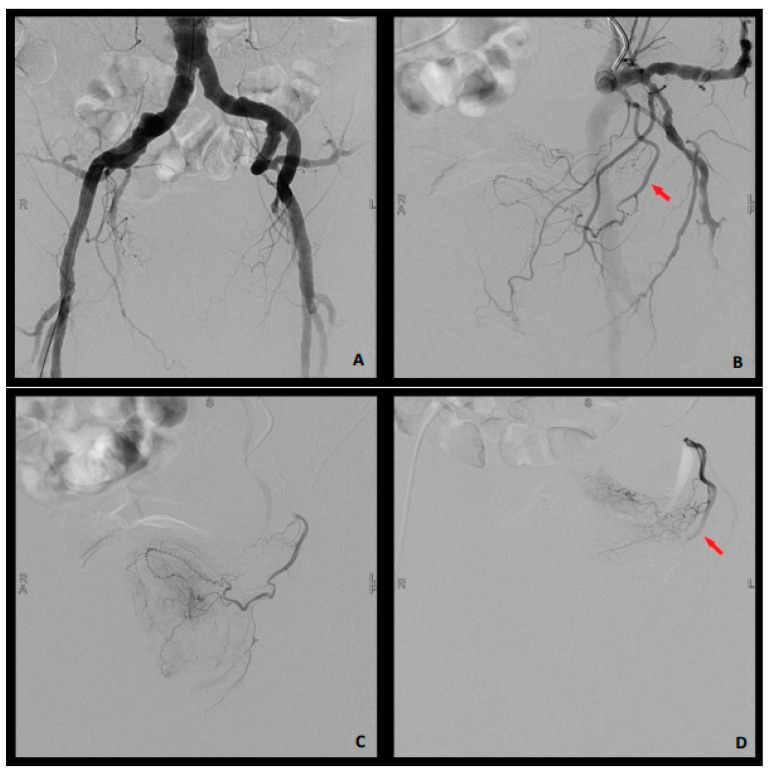
Basic steps of PAE procedure: pelvic angiography with a power injector (**A**), left side internal iliac artery, prostatic artery visible (arrow) (**B**), angiography with microcatheter of left prostatic artery with half of the lobe enhanced (**C**), post PAE angiography, contrast medium stasis in the prostatic artery (**D**).

An enrolled patient was usually admitted to the hospital on the morning of the day of the embolization procedure. A 16 F indwelling catheter was inserted into the bladder. The balloon of the indwelling catheter was filled with a 50/50 contrast medium and saline mix. We used this technique to see where the neck of the bladder is and to better visualize the prostatic gland. Before being transported to the interventional radiology suite, the patient was administered non-steroid anti-inflammatory drugs to manage the pain during the embolization (usually patients feel no to mild pain) and was advised to take it the following days after the procedure if necessary. No antibiotics were used. PAE procedure was performed using GE Innova 4100 system. A nonionic contrast medium was used (Visipaque 320 mg/mL). Intervention was performed under local anesthesia (Procaine 1% 5 mL) using right femoral access. A 5 F vascular sheath was introduced to the common femoral artery. A 5 F flush catheter (pigtail type) was placed in the distal aorta and initial pelvic angiography was performed (frontal plane, 0°) with a power injector (30 mL of contrast medium, 15 mL/s injecting rate) to evaluate the anatomy of the internal iliac arteries and their branches. A 5 F Cobra-shaped catheter was used to perform the selective angiography of the anterior division of the left internal iliac artery (ipsilateral oblique projection, 30–40°) to better visualize the origin of the left prostatic artery, which was then super selectively catheterized with 1.9 F microcatheter. The tip of the microcatheter was placed as distally into the prostatic artery as safely possible, trying to avoid vasospasm from the catheter or guiding wire. If any arterial collaterals to surrounding organs were suspected, a rotational cone-beam CT angiography was performed, and, if confirmed, those arteries were embolized with microcoils to prevent non-target embolization. A two-syringe system with spherical 400 µm in size embolization particles was prepared. Using a 2 mL syringe, the embolization material was slowly injected under fluoroscopy guidance. The endpoint of embolization was near stasis in the prostatic artery (Figure 1). A right internal iliac artery was accessed through the same right femoral access using a Simmons or Cobra shape catheter, selective ipsilateral oblique angiography was performed in the anterior division, microcatheter was placed and embolization was performed in the same manner as on the left side.

### 2.3. Post-Procedural Management and Complications

Post-PAE patients remained in the hospital till the next day and had their indwelling catheters left during this period to prevent acute urinary retention caused by post-procedural prostate tissue swelling. Appropriate hydration and pain management (if needed) were administered. 

Minor post-procedural complications such as injection site hematoma, reaction to contrast material, dysuria, pain after the procedure, urinary tract infection, hematuria, hematospermia, and acute urinary retention, and major complications such as non-targeted embolization (ischemia to the bladder wall, penis, rectum, seminal vesicles, surrounding tissues), vessel rupture due to catheterization, and radiodermatitis were also evaluated [22].

### 2.4. Technical Outcomes 

The PAE procedure is considered technically successful if at least one side of the prostatic gland is embolized. Unilateral embolization can be an endpoint of the procedure if the branches of pelvic arteries are very tortuous and it is technically difficult and almost impossible to selectively catheterize them. Another reason for unilateral embolization is advanced atherosclerosis—one of the prostatic arteries can be occluded and the ipsilateral side of the prostatic gland obtains the blood supply from the collaterals. 

Based on similar studies [8,12,23,24,25], we chose the criteria to assume that the procedure was clinically successful if the IPSS was reduced by ≥25%, and Qmax increased by at least 5 mL/s. We chose not to consider clinical success or failure based on the PV because some studies imply only a poor correlation between the reduction of PV and the clinical outcome [23]. Post-procedural complications were divided into minor, required no ambulatory treatment, and major, resulting in hospitalization or additional intervention.

### 2.5. Statistical Methods 

Statistical calculations were performed using SPSS v.29 software (IBM). Variables were checked for normality using the Shapiro–Wilk test. Descriptive statistics were used to evaluate the data: means, statistical deviations, median, interquartile deviation, confidence intervals, minimum and maximum values, and statistical analysis for dependent samples was also applied. Measured parameters (PV, Qmax, PVR, IPSS, QoL, IIEF-5 scores) at 2 months and 6 months follow-up were compared to baseline using paired Wilcoxon signed-rank tests and for 3 groups these parameters were evaluated using Friedman signed-rank test. A *p*-value <0.05 was considered statistically significant.

## 3. Results

The baseline characteristics of PAE patients are shown in Table 1. At baseline, the age of the male (N = 70) subjects was 74 ± 9.6 years with a median of 73.8, but fluctuated from 53 to 90 years. The mean prostate volume among the investigated males (N = 66) was almost 111 mL, with a median of 106 and IQR range from 77.8 to 140, and the Qmax among males (N = 37) was only close to 7.7 mL/s with a median of 6.9 and IQR range from 4.75–10.5. The average post-void residual volume in the bladder of the male (N = 37) subjects was found to be 107.6 mL, but it ranged from 0 to 350 mL. The IPSS score mean among male (N = 37) patients was 21.3 points, which shows severely symptomatic disease and the mean value of the QoL score in males (N = 66) was 4.53 points. It was determined that the value of the IIEF-5 questionnaire score among investigated males (N = 66) was almost 1.8 points, which shows severe erectile dysfunction. The average value of the PSA level in the studied men (N = 48) was 5.8 ng/mL with a median of 4.1 ng/mL and IQR was 2.8–7.4 ng/mL. In another study, patients (N = 29) had a cystostomy catheter and were completely unable to urinate on their own (no Qmax, PVR, or IPSS scores were measured). Two months after the prostatic artery embolization procedure, only 9 (31.0%) out of 29 patients had residual cystostomy catheters, and six months after the procedure, only 4 (13.8%) out of 29 patients had cystostomy catheters left. 

When evaluating the frequencies of body mass index (BMI), it was determined that 4.5% had too low a BMI. We found that 39.4% of the examined persons were overweight, five (7.6%) were obese, and almost half (48.5%) of the subjects had a normal BMI. The vast majority (80.0%) of the examined male patients had erectile dysfunction. When assessing harmful behavioral lifestyle factors, it was found that 40.9% of the studied men smoked, but only 13.6% consumed alcoholic beverages.

Technical success (bilateral and unilateral embolization) was 63/70 (90.0%), and 6/63 (9.5%) of those patients who underwent unilateral embolization due to atherosclerotic damaged arteries or anatomical difficulties. In one patient (1.43%) with severe pelvic arteries tortuousness, embolization was not performed because placing a diagnostic catheter into the anterior division of the internal iliac artery on both sides was technically impossible. Five patients (7.94%) had collateral vessels coiling to prevent non-target embolization. In these patients, the collateral vessel was identified as connecting the prostatic artery to the rectal artery branches. Two patients not satisfied with the outcome of embolization had TURP performed 1 month after the PAE. 

### 3.1. Follow-Up 

Out of 63 technically successful patients, we already have 59 (93.7%) patients with 2 and 51 (81.0%) patients with 6 months of follow-up data. On both post-procedural follow-up visits, prostate volume (PV) with transrectal ultrasound, peak urinary flow (Qmax), post-void residual volume (PVR) with transabdominal ultrasound, international prostate symptom score (IPSS), quality of life score (QoL) and international index of erectile function (IIEF) were measured. Overall, 29 of 66 (25.8%) embolized patients had cystostomy catheters prior to PAE due to chronic retention. These twenty-nine patients are excluded from further statistical calculations, and their results are presented separately: 2 months (n = 20) after prostatic artery embolization, the mean Qmax velocity was 15.0 mL/s, PVR was 47.5 mL, and IPSS was 12.5 points, while 6 months (N = 25) after the procedure, the corresponding indicators were as follows: 18.1 mL/s, 44.2 mL, and 9.3 points. 

### 3.2. Clinical Outcomes 

After the PAE, BPH-related LUTS and the majority of measured parameters improved statistically significantly (Table 2). 

After 2 months of PAE, in investigated males (N = 59), the PV significantly decreased by 30.2% from the baseline level with a mean of 77.4 ± 29.0 mL (95% PI 69.9–85.0) (*p* < 0.0001), the Qmax significantly increased by 126.1% from the baseline with a mean of 15.0 ± 4.5 mL/s (95% PI 13.7–16.3) (*p* < 0.0001), the PVR significantly decreased by 45.9% from the baseline with a mean of 46.9 ± 38.2 mL (95% PI 36.1–57.8) (*p* < 0.0001), the IPSS score significantly decreased by 33.3% from the baseline with a mean of 12.6 ± 5.5 (95% PI 11.0–14.2) (*p* < 0.0001), the QoL score significantly decreased by 60.0% from the baseline with a mean of 2.6 ± 1.2 (95% PI 2.3–3.0) and the IIEF-5 score was without significant changes. 

After 6 months of PAE, in investigated males (N = 51), the PV significantly decreased by 39.6% from the baseline level with a mean of 65.1 ± 21.3 mL (95% PI 59.1–71.1) (*p* < 0.0001), the Qmax significantly increased by 171.0% from the baseline with a mean of 18.1 ± 5.3 mL/s (95% PI 16.5–19.6) (*p* < 0.0001), the PVR significantly decreased by 47.1% from the baseline with a mean of 464.4 ± 34.9 mL (95% PI 34.2–54.7) (*p* < 0.0001), the IPSS score significantly decreased by 57.1% from the baseline with a mean of 9.3 ± 4.9 (95% PI 7.8–10.7) (*p* < 0.0001), the QoL score significantly decreased by 60.0% from the baseline with a mean of 1.8 ± 1.3 (95% PI 1.5–2.2) and the IIEF-5 score was without significant changes. The PSA levels also significantly decreased by 28.4% six months after the PAE.

After performing the PSA density (PSAD) evaluation, it was found that the average value of PSAD before and after the intervention in the studied patients (N = 35) did not differ significantly and was 0.051 ± 0.042 and 0.057 ± 0.042, respectively (*p* = 0.24).

During the follow-up, we observed ten patients with post-procedural complications: nine minor and one major events. Those are discussed in more detail in Section 4.

**Figure 2:**
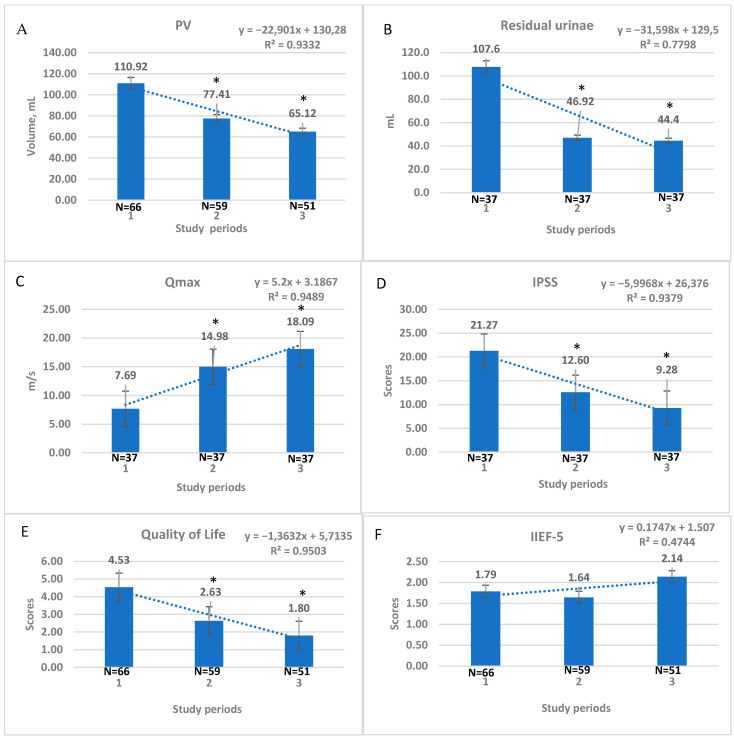
Mean with 95% CI (**A**) PV (mL), (**B**) PVR (mL), (**C**) Qmax (mL/s), (**D**) IPSS (score), (**E**) QoL (score), and (**F**) IIEF-5 (score) at baseline (1) and at 2 months (2) and 6-month (3) follow-up. * *p* < 0.05, comparing data at baseline to follow-up at 2 and 3 study periods.

The mean values of the baseline and follow-up data are summarized in Figure 2.

## 4. Discussion

Patient’s with BPH-related symptoms usually first receive pharmacotherapy, which mostly includes 5α reductase inhibitors and α blockers. When medical treatment is insufficient, surgical treatment is usually the next option. The gold standard for BPH up to 60–80 mL in volume is TURP, and for larger prostates, it is usually open prostatectomy. Both of these options can reduce the LUTS significantly. However, with surgical therapies, there are always possible limitations (comorbidities of the patient that make surgical treatment impossible and various complications that can occur after the surgery) [24,25,26,27]. In recent years, more and more studies have been published introducing PAE as an alternative treatment method for BPH [4,8,12,13,14,27,28]. Prostatic artery embolization (PAE) is performed under local anesthesia, using the femoral approach with no or minimal pain, so it can be performed for those patients that are not suitable for surgery or general anesthesia. Also, while small-sized glands are not suitable for PAE, there is no upper limit for the prostate size for PAE. Another advantage of PAE is its low complication rate, with the majority being minor complications [8,12,16,23,24,29]. According to our results, 9/63 patients after PAE had minor and 1/63 had major complications: five out of nine patients had dysuria, lasting up to 2 weeks, and three out of nine had a urinary tract infection requiring antibiotic treatment. One patient returned to the ER during the first week after embolization with acute retention and was treated with an indwelling catheter for 10 days—we believe the obstruction was caused by prostate edema and swelling due to necrosis after embolization. One patient who had major complications complained of intermittent obstruction during urination; transabdominal ultrasound showed a 2–3 cm irregular mass inside the bladder. Cystoscopy was performed and a sloughing fibrin clot/necrotic mass was resected. In an article published by Ayyagari et al., the same complication is described [28]. We believe the necrotic tissue formed because of non-target embolization to the wall of the bladder, probably to the inferior vesicle artery. These kinds of complications should be avoided as the experience of interventional radiologists grows in the future. We compared the improvement of BPH-related symptoms with other authors’ results [8,12,14,19,27]. In our study at 6 months follow-up, the mean values of IPSS, QoL, IIEF, PV, and PVR improved similarly. However, the mean Qmax after PAE for our patients after 2 and 6 months improved by 15.0 mL/s and 18.1 mL/s, respectively; in comparison to the previously mentioned studies, the mean improvement was 3,07 mL/s, 6.6 mL/s, 7.8 mL/s, and 7.0 mL/s [8,12,18,19,27]. For the embolization material, we chose 400 µm spherical particles. There are several articles [23,27] debating the type and size of the embolic particle. We chose the upper size range because we think it is safer and easier to avoid non-target embolization to the bladder, rectal, or penile arteries. On the other hand, larger particles are expected to clog the artery more proximally and do not reach the distal branches in the periurethral region, where the shrinkage of the gland is mostly required. However, comparing our early results with those of other studies where different-sized particles were used, we see very similar results, which may indicate that the size of the particle is not the most important factor determining the clinical outcome. Daniel Torres et al. found similar results in their study, comparing different-sized particles in PAE [27].

As for future perspectives, we consider that one of the most important factors in PAE should be patient selection, especially the size of the prostatic gland. In this study, there were patients with higher volume prostates (>60 mL), when the prostatic arteries usually are more hypertrophied and easier to access. According to our early results, we observe a tendency that the bigger the prostate, the better the outcome, but more data are needed to statistically prove it. Literature data insufficiently describe the outcomes after PAE of smaller glands—this factor is also important for patients for whom surgical therapy is contraindicated because of comorbidities [30]. Most studies include patients with PV >80 mL [4,11,12,13]. These patients would otherwise have an open prostatectomy (OP) performed, as the volume of the gland would be too large for TURP. Thus, if the patients are suitable for both minimally invasive and surgical therapies, maybe a control group of OP would be needed to compare the outcomes and longevity of both procedures. Follow-up data from our first patients show promising results, but the limitations should be considered. Our latest follow-up was at 6 months. Although the main urological outcomes improved significantly, longer observations (1–3 years) are required to track the changes in the prostate gland over time after PAE: formation of arterial collaterals, and worsening of the symptoms are possible [31,32,33,34]. Also, a control group is needed to analyze the impact of PAE on erectile function.

### Limitations

One of the possible limitations of this study was the relatively small number of patients. However, even with the small number, quite significant results were obtained. As already mentioned, the volume of the prostate at the starting point is an important factor that can determine the obtained results. In this study, comorbidities were also not evaluated, which could lead to one or another evaluation of the patient’s quality of life. Some lifestyle factors (smoking, alcohol consumption, sexual life) and risk factors for chronic non-infectious diseases (overweight, metabolic disorders, physical inactivity, environmental toxins) could also distort the research data.

## 5. Conclusions

This study shows promising results for patients with benign prostatic hyperplasia after prostatic artery embolization. The main prostate-related parameters (PV, Qmax, PVR, IPSS) improved significantly 6 months after embolization. Studies demonstrate that this technique is not only safe for the patient, with minimal complications rate, but it also has a competitive value compared to typical gold standard surgical procedures. PAE could be especially important for comorbid patients who are not suitable candidates for surgery due to high-risk mortality and complications after TURP. More studies and especially longer follow-ups are needed to analyze the longevity of LUTS improvement after prostatic artery embolization.

## Figures and Tables

**Table 1:** Baseline characteristics of prostatic artery embolization patients.

Variables	Mean ± SD	Median	IQR	Min–Max
Age (N = 70)	74.0 ± 9.6	73.8	67.2–81.9	52.8–89.8
PV (mL) (N = 66)	110.92 ± 38.72	106	77.8–140	56–210
Qmax (mL/s) (N = 37)	7.69 ± 4.04	6.9	4.75–10.5	1.4–17.6
PVR (mL) (N = 37)	107.60 ± 84.50	85	38–167.5	0–350
IPSS (score) (N = 37)	21.27 ± 5.23	21	17.5–24.5	10–32
QoL (score) (N = 66)	4.53 ± 0.98	5	4–5	0–6
IIEF-5 (score) (N = 66)	1.79 ± 4.49	0	0–0	0–21
PSA (ng/mL) (N = 48)	5.80 ± 4.79	4.1	2.8–7.4	0.36–19

PV—prostate volume, Qmax—uroflowmetry, PVR—post-void residual volume, IPSS—International Prostate Symptom Score, QoL—quality of life score, IIEF-5—International Index of Erectile Function short form, PSA—prostate antigen, IQR—interquartile range.

**Table 2:** The variables of prostatic artery embolization patients at baseline and after 2 and 6 months of follow-up.

Variables	Mean ± SD	95% CI		Median	Median Changes from Baseline	*p* Values by Friedman	*p* Values by Wilcoxon
		Lower	Upper		%		
PV						<0.000	
Baseline (N = 66)	110.92 ± 38.72	101.40	120.44	106			
2 months (N = 59)	77.41 ± 28.96	69.86	84.95	74	−30.2		<0.000
6 months (N = 51)	65.12 ± 21.32	59.12	71.12	64	−39.6		<0.000
Qmax						<0.000	
Baseline (N = 37)	7.69 ± 4.04	6.35	9.04	6.9			
2 months (N = 37)	14.98 ± 4.48	13.70	16.25	15.6	+126.1		<0.000
6 months (N = 37)	18.09 ± 5.27	16.54	19.63	18.7	+171.0		<0.000
PVR						<0.000	
Baseline (N = 37)	107.60 ± 84.50	79.42	135.77	85			
2 months (N = 37)	46.92 ± 38.22	36.06	57.79	46	−45.9		<0.000
6 months (N = 37)	44.40 ± 34.89	34.16	54.65	45	−47.1		<0.000
IPSS						<0.000	
Baseline (N = 37)	21.27 ± 5.23	19.53	23.01	21			
2 months (N = 37)	12.60 ± 5.51	11.03	14.17	14	−33.3		<0.000
6 months (N = 37)	9.28 ± 4.88	7.84	10.71	9	−57.1		<0.000
QoL						<0.000	
Baseline (N = 66)	4.53 ± 0.98	4.29	4.77	5			
2 months (N = 59)	2.63 ± 1.23	2.31	2.95	2	−60.0		<0.000
6 months (N = 51)	1.80 ± 1.27	1.45	2.16	2	−60.0		<0.000
IIEF-5						0.12	
Baseline (N = 66)	1.79 ± 4.49	0.68	2.89	0			
2 months (N = 59)	1.64 ± 3.77	0.66	2.63	0			0.5
6 months (N = 51)	2.14 ± 4.57	0.85	3.42	0			0.3
PSA							
Baseline (N = 48)	5.80 ± 4.79	4.41	7.19	3.7			
2 months							
6 months (N = 28)	3.79 ± 3.21	2.54	5.03	2.65	−28.4		0.03

## Data Availability

Data are available upon contact with authors.

## Abbreviations

BPH: benign prostate hypertrophy; PAE: prostatic artery embolization; LUTS: lower urinary tract symptoms; TURP: transurethral resection of the prostate; Qmax: maximum urinary flow rate; IPSS: International Prostate Symptom Score; QoL: Quality of Life; IIEF-5: International Index of Erectile Function; PV: prostatic volume; PVR: post-void residual volume; PSA: prostate-specific antigen.
